# The role of whole-brain radiotherapy (WBRT) in primary central nervous system lymphoma: is it an alternative to ASCT for consolidation following HD-methotrexate based induction in low-income settings?

**DOI:** 10.1186/s13014-022-02142-y

**Published:** 2022-10-22

**Authors:** Luís Alberto de Pádua Covas Lage, Vinícius Araújo Soares, Thales Dalessandro Meneguin, Hebert Fabrício Culler, Cadiele Oliana Reichert, Mayara D’Auria Jacomassi, Diego Gomes Cândido Reis, Maria Cláudia Nogueira Zerbini, Renata de Oliveira Costa, Vanderson Rocha, Juliana Pereira

**Affiliations:** 1 Department of Hematology, Hemotherapy and Cell Therapy, Faculty of Medicine, University of São Paulo (FM-USP), São Paulo, Brazil; 2 Laboratory of Medical Investigation in Pathogenesis and Directed Therapy in Onco-Immuno-Hematology (LIM-31), University of São Paulo (USP), Cerqueira César, Avenue Dr. Enéas de Carvalho Aguiar, 155 – Ambulatory building – 1st. Floor, Room 61, São Paulo (SP), 05403-000 Brazil; 3 Department of Pathology, Faculty of Medicine, University of São Paulo (FM-USP), São Paulo, Brazil; 4 Department of Hematology and Hemotherapy, Faculty of Medical Sciences Santos (FCMS), Centro Universitário Lusíadas (Unilus), Santos, Brazil; 5 Fundação Pró-Sangue, Blood Bank of São Paulo, São Paulo, Brazil; 6 Churchill Hospital, Oxford University, Oxford, UK; 7 Hospital Alemão Osvaldo Cruz, São Paulo, Brazil

**Keywords:** Primary central nervous system lymphoma (PCNSL), Whole-brain radiotherapy (WBRT), Outcomes, Prognostic factors, Resource-constrained settings

## Abstract

**Background:**

Primary central nervous system lymphoma (PCNSL) is a rare and aggressive malignancy. Although potentially curable, its prognosis remains dismal. Its treatment is based on high-doses of methotrexate (HD-MTX) and rituximab, followed by consolidation therapy with whole-brain radiotherapy (WBRT) or autologous stem cell transplantation (ASCT). Currently, there is no consensus about the best consolidation strategy, but better outcomes with ASCT are obtained with conditioning regimens based on thiotepa, a high-cost drug with restricted use in resource-constrained settings. Latin American data on clinical outcomes, prognostic factors, and therapeutic management in PCNSL are virtually unknown.

**Methods:**

This is a retrospective, observational, and single-center study involving 47-Brazilian patients with PCNSL. We aim to assess outcomes, determine predictors of survival, and compare responses, as well as toxicities in patients consolidated with chemotherapy alone versus chemotherapy plus WBRT.

**Results:**

The median age at diagnosis was 59 years (24–88 years), and 53.1% were male. LDH ≥ UVN occurred in 44.7%, ECOG ≥ 2 in 67.6%, and 34.1% had multifocal disease. Hemiparesis was the main clinical presentation, observed in 55.3%, 51.0% had intermediate-/high-risk IELSG prognostic score, and 57.6% had an ABC-like phenotype by IHC. With a median follow-up of 24.4 months, estimated 5-year OS and PFS were 45.5% and 36.4%, respectively. Among 40 patients treated with HD-MTX-based induction, estimated 2-year OS was 85.8% for those consolidated with WBRT plus HIDAC versus only 41.5% for those consolidated with HIDAC alone (p < 0.001). Hematologic and non-hematologic toxicities were not significant, and severe cognitive impairment occurred in only 6.3% (3/47) of cases, all of them treated with WBRT. Age < 60 years, Hb ≥ 120 g/L and WBRT consolidation were associated with increased OS, however, LDH ≥ UVN, hypoalbuminemia, ECOG ≥ 2, Karnofsky PS < 70 and intermediate-/high-risk Barcelona score were associated with decreased OS.

**Conclusion:**

Combined consolidation therapy (CCT) based on WBRT plus HIDAC was associated with increased OS in PCNSL compared to isolated consolidation therapy (ICT) based on HIDAC alone. Here, severe late neurotoxicity was uncommon with this approach. These data suggest that WBRT may be an effective and safe alternative to ASCT for consolidation therapy in PCNSL, particularly in resource-constrained settings, where access to thiotepa for pre-ASCT conditioning is not universal.

## Introduction

Primary central nervous system lymphoma (PCNSL) includes different subtypes of non-Hodgkin's lymphomas (NHL) restricted to the central nervous system (CNS), involving structures such as the brain, cerebellum, brainstem, spinal cord, eyes, and leptomeninges [44]. By definition, lymphomas arising from the dura mater, intravascular diffuse large B-cell lymphoma (iDLBCL), those associated with immunosuppression [such as AIDS-related lymphomas (ARL) and post-transplant lymphoproliferative disorders (PTLD)] and lymphomas with evidence of systemic involvement should not be classified as PCNSL [9, 44]. Although low-grade B-cell lymphomas and mature T-cell lymphomas may primarily involve the CNS, more than 95% of PCNSL cases show diffuse large B-cell lymphoma (DLBCL) histologic pattern [9, 44].

PCNSL is rare, accounting for 4% of CNS tumors and 4–6% of extranodal lymphomas [18, 44]. Its incidence is 0.4 cases/100,000 people per year in the United States, with a slight predominance in males and with a median age of 60 years at diagnosis [18, 29, 44]. Despite its aggressive biological behavior, these tumors are potentially curable, especially when it affects young patients [9, 18].

Although the biological advances obtained in the last decade, many caveats addressing the PCNSL pathogenesis persist. One of them is related to the normal counterpart of the malignant cells. As there is no resident or native lymphoid cells within the CNS it has been speculated that the malignant cell of the PCNSL could be derived from the local malignant transformation of B-lymphocytes transiting through the CNS or even from a systemic transformation of B-cells with tropism for the CNS [30]. In contrast to PCNSL associated with immunosuppression, where the Epstein-Barr virus (EBV) plays a key role in the tumor development, in immunocompetent patients there is no evidence of EBV-DNA in neoplastic cells [8, 28, 36]. Recent studies involving whole-exome sequencing (WES) have found recurrent mutations driving the PCNSL pathogenesis. It was identified biallelic losses of *CDKN2A* and somatic mutations in the *MYD88*, *CD79b* and *TBL1XR1* genes [7, 17, 32]. Similarly, constitutive activation of the B-cell receptor (BCR), Toll-like receptor and NF-kB signaling pathway have been implicated in the pathogenesis of PCNSL [4].

Over the last five decades, there has been a slight increase in PCNSL survival, with a median overall survival (OS) of 12.5 months in the 1970s, increasing to just 26 months in the last ten years [9]. Although clinical outcomes remain poor, its prognosis is markedly heterogeneous.

Therefore, in 2003, the *International Extranodal Lymphoma Study Group* (IELSG) proposed a score of prognosis based on five variables, including age ≥ 60 years, performance status by *Eastern Cooperative Oncology Group* (ECOG) ≥ 2, serum lactic dehydrogenase (LDH) ≥ normal values (UVN), deep brain involvement and high protein concentration in the cerebrospinal fluid (CSF) (≥ 40 mg/dl). Patients with 0–1 factors were categorized in low-risk with an estimated 2-year OS of 80% and those with 4–5 factors were classified as high-risk, with an estimated 2-year OS of only 15% [13]. Subsequently, other prognostic systems were proposed. However, these scores were based on case series treated in developed countries [3, 5]. The Latin American data concerning outcomes and prognostic factors of PCNSL patients are very scarce [37].

The PCNSL management involves an initial phase of induction followed by a consolidation therapy. The induction phase has high-dose methotrexate (HD-MTX) as backbone, associated with others cytotoxic agents able to cross the blood–brain barrier, as cytarabine, vinca alkaloids, alkylating agents and the anti-CD20 monoclonal antibody rituximab [1, 14, 16, 31, 33, 35]. The whole-brain radiotherapy (WBRT) or autologous hematopoietic stem cell transplantation (ASCT) are the main strategies for consolidation and are used to ensure long-term remissions and reduce early-relapses [9, 18, 20]. Currently, there is no consensus for choose ASCT or WBRT to consolidate the responses obtained after HD-MTX-based induction in PCNSL patients [38, 12, 14, 19, 22].

The long-term effects of WBRT are fearsome, but ASCT is not a risk-free therapy. Better results with ASCT were obtained based on the use of thiotepa as the centerpiece of the conditioning regimen. However, thiotepa is not available in our country and it is a high-cost drug, limiting it use in low-income countries [12, 25, 45]. It is noteworthy that thiotepa is not an affordable and universal drug to be used in Brazil, making the consolidation with ASCT for PCNSL an exception in our country, especially in the context of public health. The same situation occurs in other areas of the world outside of North America and Western Europe.

Based on this premise, the current study aimed to describe the clinical, laboratory and epidemiological characteristics, and outcomes of PCNSL immunocompetent patients treated in the largest cancer center of Latin America. We also compared consolidation with WBRT plus chemotherapy versus chemotherapy alone in our cohort of patients treated in a real-life setting.

## Patients and methods

### Study design and ethical issues

This is a retrospective and observational study, performed in a single-center, at the Instituto do Câncer do Estado de São Paulo/Hospital das Clínicas, Medicine School, University of São Paulo, Brazil. It was approved by the local Ethics Committee in 2021 (number: 48394721.3.0000.0068). The waiver of the Free and Informed Consent Term was obtained. All clinical, epidemiological and laboratory data were taken from electronic medical records.

### Patients

Initially, were identified 55 patients with a biopsy-proven diagnosis of PCNSL treated at our institution from January 2000 to December 2021. Eligibility criteria included age ≥ 18 years, DLBCL confirmed by histology and restricted to CNS (PCNSL). After applying the eligibility criteria, 47 patients were included in the final analysis. Four patients were excluded because they were related to Immunosuppression (ARL [N = 3] and PTLD [N = 1]). One case presented histology other than DLBCL, one had systemic involvement, and in two cases there were no clinical data available.

Clinical, epidemiological, comprehensive laboratory data and histological features accessed at diagnosis included age, gender, ethnicity, performance status by ECOG, Ann Arbor/Cotswold clinical stage, *International Extranodal Lymphoma Study Group* (IELSG), *Memorial Sloan Kettering Cancer Center* (MSKCC), and *Nottingham/Barcelona* prognostic scores for PCNSL. The main clinical signs and symptoms, disease extension (unifocal versus multifocal), involvement of deep brain structures, orbit/eyeball, and cranial nerves impairment were also assessed. LDH values, β2-microglobulin (β2MG), serum albumin, whole blood count and CSF protein were recorded. DLBCL classification based on IHC parameters, according to Hans algorithm were also captured [21].

Date of diagnosis, complete response (CR), relapse and disease progression, the first and last cycle of chemotherapy and radiotherapy, date and cause of death and date of last follow-up were taken to assess overall response rate (ORR), overall survival (OS), progression-free survival (PFS), and progression of disease within 24 months of the end of up-front therapy (POD-24). At diagnosis, all patients performed complete blood count, comprehensive biochemical tests, including renal and liver function, LDH, β2-microglobulin and serology for HIV, hepatitis B and C.

Staging was carried out by computed tomography (CT) scans of the neck, chest, abdomen, and pelvis or by CT with positron emission with 18-fluorodeoxyglucose (18-FDG-PETCT) and unilateral bone marrow biopsy to exclude systemic involvement. All patients performed brain magnetic resonance imaging (MRI) and CSF examination with oncotic cytology, as well as ultrasound of the testes and ophthalmologic examination with fundoscopy and slit lamp.

### Therapeutic management, response assessment and follow-up

Most patients were treated with HD-MTX, vincristine and procarbazine (MPV) [1, 31] and some cases received MPV plus rituximab (R-MPV) [35]. In brief, an induction phase consisted of methotrexate 3500 mg/sqm I.V. on D1, D15, D30, D45 and D60 plus rescue with calcium folinate, vincristine 1.4 mg/sqm [max 2.0 mg] I.V. on D1, D30 and D60 and procarbazine 100 mg/sqm/day P.O. on D1-D7 and D45-D51 ± rituximab 375 mg/sqm I.V. on D1, D15, D30, D45 and D60. All patients received prophylactic and/or therapeutic oral laser and urine alkalization with sodium bicarbonate was indicated to prevent or minimize HD-MTX kidney toxicity. Cerebrospinal fluid puncture with intrathecal (IT) MADIT application (methotrexate 12 mg/dose, cytarabine 60 mg/dose and dexamethasone 2 mg/dose) was performed on D1, D15, D30, D45 and D60, unless if contraindicated. Patients with lymphomatous meningitis received MADIT twice weekly until CSF clearance, followed by MADIT weekly for four consecutive weeks.

After induction, patients were submitted to brain MRI and CSF analysis to verify the interim response according to international criteria for PCNSL evaluation [2]. Patients under < 60 years that obtained CR or PR underwent combined consolidation therapy (CCT) with WBRT at dose of 45 Gy (25 fractions of 180 Gy) followed by two cycles of HIDAC (high-dose cytarabine)—3000 mg/sqm I.V. on D1 with a 30-day interval. To minimize long-term toxicities related with WBRT, patients above ≥ 60 years in CR were consolidated with only two cycles of cytarabine at a dose of 1500 mg/sqm I.V. on D1 with an interval of 30 days. Patients above ≥ 60 years with PR received CCT, based on WBRT (45 Gy) followed by two cycles of cytarabine 1500 mg/sqm I.V. on D1 every 30 days. The last group underwent to brain MRI and CSF analysis at the end of the second cycle of HIDAC to assess the final response. Very elderly (≥ 80 years) patients, frail (ECOG ≥ 3) or with Charlson index > 2 were considered for palliative care with WBRT or MADIT plus dexamethasone. No patient was consolidated with ASCT.

**Fig. 1:**
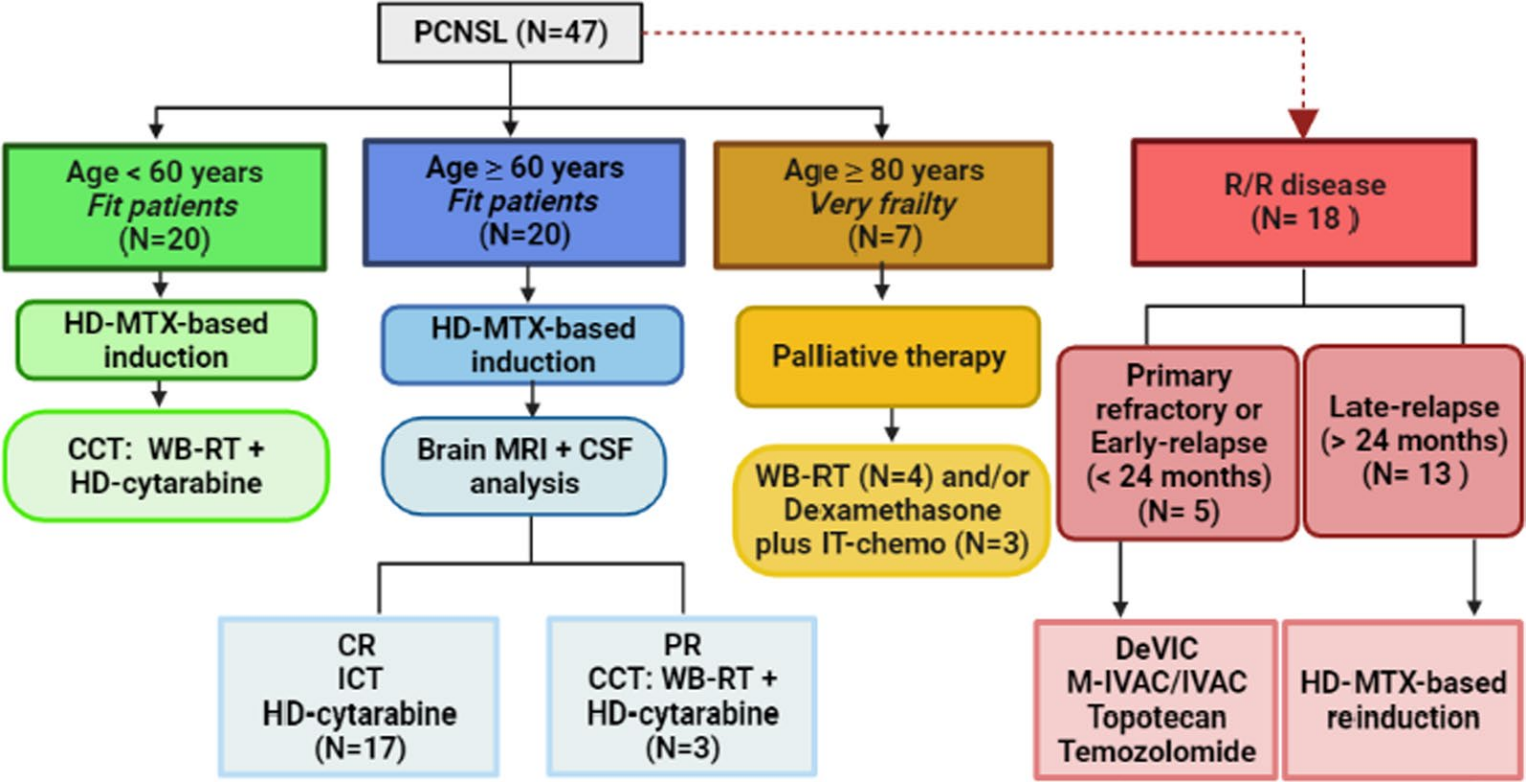
Therapeutic approach of the 47-Brazilian patients with PCNSL included in the study. *PCNSL* primary central nervous system lymphoma, *HD-MTX* high-dose methotrexate, *CCT* combined consolidation therapy, *HD-cytarabine* high-dose cytarabine, *MRI* magnetic resonance imaging, *CSF* cerebrospinal fluid, *CR* complete response, *PR* partial response, *WBRT* whole-brain radiotherapy, *IT* intratecal, *R/R* relapsed/refractory disease, *DeVIC* dexamethasone, etoposide, ifosfamide and carboplatin, *M-IVAC* methotrexate, ifosfamide, etoposide, cytarabine. ***Among the 13 patients with late-relapse, only 5 cases received HD-MTX-based rescue therapy. The remaining 8 patients died before a new treatment

For salvage, fit patients relapsed after 24 months from the up-front therapy were rescued with HD-MTX-based regimens, and primary refractory and early-relapsed cases received different regimens, such as IVAC/M-IVAC (methotrexate/ifosfamide, etoposide, cytarabine), DeVIC (dexamethasone, etoposide, ifosfamide and carboplatin), topotecan or temozolomide. Clinical follow-up was done every 3 months for the first two years after CR, every 6 months for the third and fourth year, and annually after the fifth year. Figure 1 summarizes the therapy used for the 47-Brazilian PCNSL patients.

### Histopathology and immunohistochemistry

All cases were submitted to centralized histopathological review by two experts in Hematopathology and categorized as PCNSL according to the World Health Organization Classification for Hematopoietic and Lymphoid Tissue Neoplasms (WHO-2016) [44]. The tissue biopsies obtained at diagnosis were keep in formalin-fixed paraffin-embedded (FFPE) material. FFPE sections of 2 µm were displayed on silanized slides and stained with Hematoxylin & Eosin for initial analysis. Immunohistochemistry was carried out with monoclonal antibodies CD45 (Dako, 2B11 + PD7/26, 1/2000), pan-B CD20 (Dako, L26, 1/1000), pan-T CD3 (Dako, F7.2.38, 1/500), proliferative marker Ki-67 (Dako, J55, 1/1600), CD10 (Novocastra, S6C6, 1/2000), BCL-6 (Abcam, EPR11410-43, 1/500) and MUM-1/IRF-4 (Abcam, EPR5653, 1/500). Pan-cytokeratin AE-1/AE-3 (LSBio, AE1 + AE3, 1/100) and anti-glial fibrillary acidic protein [GFAP] (Merck, G-A-5, 1/300) were used to exclude carcinomatous metastasis and high-grade gliomas, respectively. For the immunohistochemical algorithm proposed by Hans et al. [21] to determine the cell of origin (COO) was used the markers CD10, BCL-6 and MUM-1/IRF4 [21].

### Statistical analysis

Data were showed in accordance with the variables evaluated. Categorical variables were presented in absolute (N) and relative (%) values. Numerical variables were presented as measures of central tendency (median), dispersion (interquartile range, IqR) and position. Analysis of overall survival (OS) and progression-free survival (PFS) were performed using the Kaplan–Meier (KM) method, and the Log-Rank test was used for comparison between treatment groups. OS was considered from the date of diagnosis to death, and PFS from the date of diagnosis to disease progression, death, or last follow-up. Progression of disease within 24 months (POD-24) was considered as the percentage of cases that presented disease progression within 24 months from up-front treatment. Data were censored at the last follow-up.

Analysis to determine predictors for outcomes, including those related with OS and PFS, was performed using Cox’s semiparametric univariate method. The results were presented in Hazard Ratio (HR) and 95% Confidence Interval (95% CI). All analyzes were performed using the R-statistical software version 4.1.3 for Windows and a *p*-value ≤ 0.05 was assigned as statistically significant. Due to the relative small number of cases (N = 47) multivariate analysis could not be performed.

## Results

### Clinical, epidemiological and histopathological findings

Clinical, laboratory, epidemiological and pathological features of the 47 patients were displayed in Table 1. The median age was 59 years (range: 24–88 years), 53.1% (25/47) were male, and 76.6% (36/47) were caucasian. LDH ≥ UVN was observed in 44.7% (21/47) and β2-microglobulin ≥ UVN in 62.1% (29/47). Low albumin levels (inferior to 3.5 g/dL) were found in 20% (9/47) of cases, ECOG ≥ 2 in 67.6% (32/47) and Karnofsky performance status (KPS) < 70 in 38.3% (18/47). Unifocal disease occurred in 65.9% (31/47) and 34.1% (16/47) had multifocal disease, characterized by more than one CNS parenchymal lesion or meningeal involvement. Single or multiple expansive parenchymal lesions were detected in 80.8% (38/47) and lymphomatous meningitis in 19.2% (9/47).

**Table 1:** Clinical, laboratory and pathological features of the 47-Brazilian patients with PCNSL

Characteristic	N = 47 (N, %)
Median age (years)	59.8(24.9–88.1)
Male gender	25 (53.1)
Caucasian	36 (76.6)
LDH ≥ UVN	21 (44.7)
β2-microglobulin ≥ UVN	29 (62.1)
Albumin < 3.5 g/dL	09 (19.2)
ECOG ≥ 2	32 67.6
Karnofsky < 70	18 (38.3)
Multifocal disease	16 (34.1)
CSF with positive oncotic citology	09 (19.2)
*Clinical* Hemiparesis or hemiplegia Seizures Confusional state Cranial nerve impairment	23 (55.3) 05 (10.6) 15 (31.9) 01 (2.2)
Deep brain involvement	21 (44.7)
CSF protein > 40 mg/dL	29 (61.7)
Prognostic scores intermediate and high-risk IELSG MSKCC Nottinghan/Barcelona	24 (51) 19 (40.4) 17 (36.1)
Anemia	09 (19.2)
Neutrophilia	18 (38.3)
Lymphopenia	03 (6.3)
Monocytosis	01 (2.2)
Thrombocytopenia	0 (0)
GC-like subtype	14/33 (42.4)
non-GC subtype	19/33 (57.6)

*LDH* latic dehydrogenase, *UVN* Upper value of normality, *ECOG* Eastern Cooperative Oncology Group scale, *CSF* cerebrospinal fluid, *PCNSL* primary central nervous system lymphoma, *IELSG* International Extranodal Lymphoma Study Group, *MSKCC* Memorial Sloan Kettering Cancer Center, *GC-like* germinal center-like phenotype, *COO* cell of origin, *non-GCB* non- germinal center phenotype

Regarding to clinical presentation, 55.3% (26/47) of patients had hemiparesis or hemiplegia, 31.9% (15/47) presented consciousness impairment, 10.6% (5/47) had seizures, and 2.2% (1/47) showed cranial nerve impairment. Deep brain Involvement was observed in 44.7% (21/47) of patients and high level of protein > 40 mg/dl in CSF in 61.7% (29/47). Intermediate-risk/high-risk of IELSG, MSKCC and Nottingham/Barcelona scores occurred in 51.0% (24/47), 40.4% (19/47), and 36.1% (17/47) of cases, respectively.

The median of hemoglobin, neutrophil, lymphocyte, monocyte, and platelet counts were 140 g/L, 5.6 × 10^9^/L, 1.7 × 10^9^/L, 0.6 × 10^9^/L, and 227 × 10^9^/L, respectively. Hb < 120 g/L, neutrophilia above 7.0 × 10^9^/L, lymphopenia inferior to 0.9 × 10^9^/L, monocytosis higher than 1.0 × 10^9^/L and thrombocytopenia inferior to 100 × 10^9^/L occurred in 19.2% (9/47), 38.3% (18/47), 6.3% (3/47), 2.1% (1/47), and 0% (0/47), respectively. The Hans’s algorithm could be established in 33/47 (70.3%) cases; 42.4% (14/33) were germinal-center like (GC-like) and 57.6% (19/33) non-germinal center (ABC-subtype, *activated B-cell*).

### Therapeutic modalities and toxicities

Among the 47 patients with PCNSL included in the study, 48.9% (23/47) received induction chemotherapy based on HD-MTX followed by WBRT plus high-dose cytarabine (HIDAC), and 36.1% (17/47) experienced HD-MTX-based induction followed by single consolidation chemotherapy (ICT) with HIDAC. Four patients (8.5%) received palliative therapy with WBRT alone and three cases (6.3%) did not received any systemic anticancer therapy, but only dexamethasone and/or intrathecal chemotherapy. WBRT was used for curative or palliative intention in 57.4% (27/47) of patients and 21.2% (10/47) received more than one line of anticancer therapy. No one patient was consolidated with ASCT (Table 2).

**Table 2:** Treatment modalities and adverse events profile

Characteristic	N = 47 (N, %)
*Up-front therapy* MPV ± R followed by WBRT + HIDAC MPV ± R followed by HIDAC WBRT isolated *Steroids ± intrathecal chemotherapy*	23 (48.9) 17 (36.1) 04 (8.5) 03 (6.5)
Palliative or curative WBRT	27 (57.4)
≥ 1 line of therapy	10 (21.2)
ASCT consolidation	0 (0)
Infection	13 (27.6)
Febrile neutropenia	06 (12.7)
G3/G4 neutropenia	08 (17.0)
G3/G4 thrombocytopenia	06 (12.7)
G3/G4 acute kidney injury	08 (17.0)
G3/G4 mucositis	05 (10.6)
Dementia	03 (6.3)

*MPV* methotrexate, procarbazine and vincristine, *R* rituximab, *WBRT* whole brain radiotherapy, *HIDAC* high-dose cytarabine, *ASCT* autologous hematopoietic stem cell transplantation, *G3/G4* grade 3 or 4 toxicities (CTC version 4.0.)

During the up-front therapy, 27.6% (13/47) of patients presented documented infection, 12.7% (6/47) had febrile neutropenia, 17.0% (8/47) G3/G4 neutropenia, and 12.7% (6/47) G3/G4 thrombocytopenia. Non-hematological toxicities were unusual, with acute kidney injury observed in 17.0% (8/47) and G3/G4 mucositis in 10.6% (5/47) of cases. No one severe leukoencephalopathy case was documented, and 6.3% (3/47) developed severe dementia, all of them received WBRT. Specific neurological scales to detect subclinical or mild cognitive damage were not applied and these abnormalities could not be assessed.

### Outcomes

Among the 27 patients submitted to WBRT, 23/27 had a curative intention and 4/27 had palliative purpose (WBRT alone). The ORR for the whole cohort was 59% (26/44) (95% CI: 48.8–71.2%). The complete response (CR) was obtained in 50% (22/44) (95% CI: 37.8–62.2%) and partial response (PR) in 9% (4/44) (95% CI: 1.9–16.1%) of patients. Primary refractory disease occurred in 11% of cases (5/44) (95% CI: 3.2–18.7%), 30% (13/44) (95% CI: 18.6–41.3%) presented disease relapse, being POD-24 in 11% (5/44) (95% CI: 3.2–18.7%). The overall mortality rate was 62% (30/47) (95% CI: 50.3–73.6%), 47% (14/30) of deaths were associated with disease progression, 16.7% (5/30) were secondary to infectious complications, and in 36.3% (11/30) of cases the causal death could not be obtained since they occurred out of our service.

**Fig. 2:**
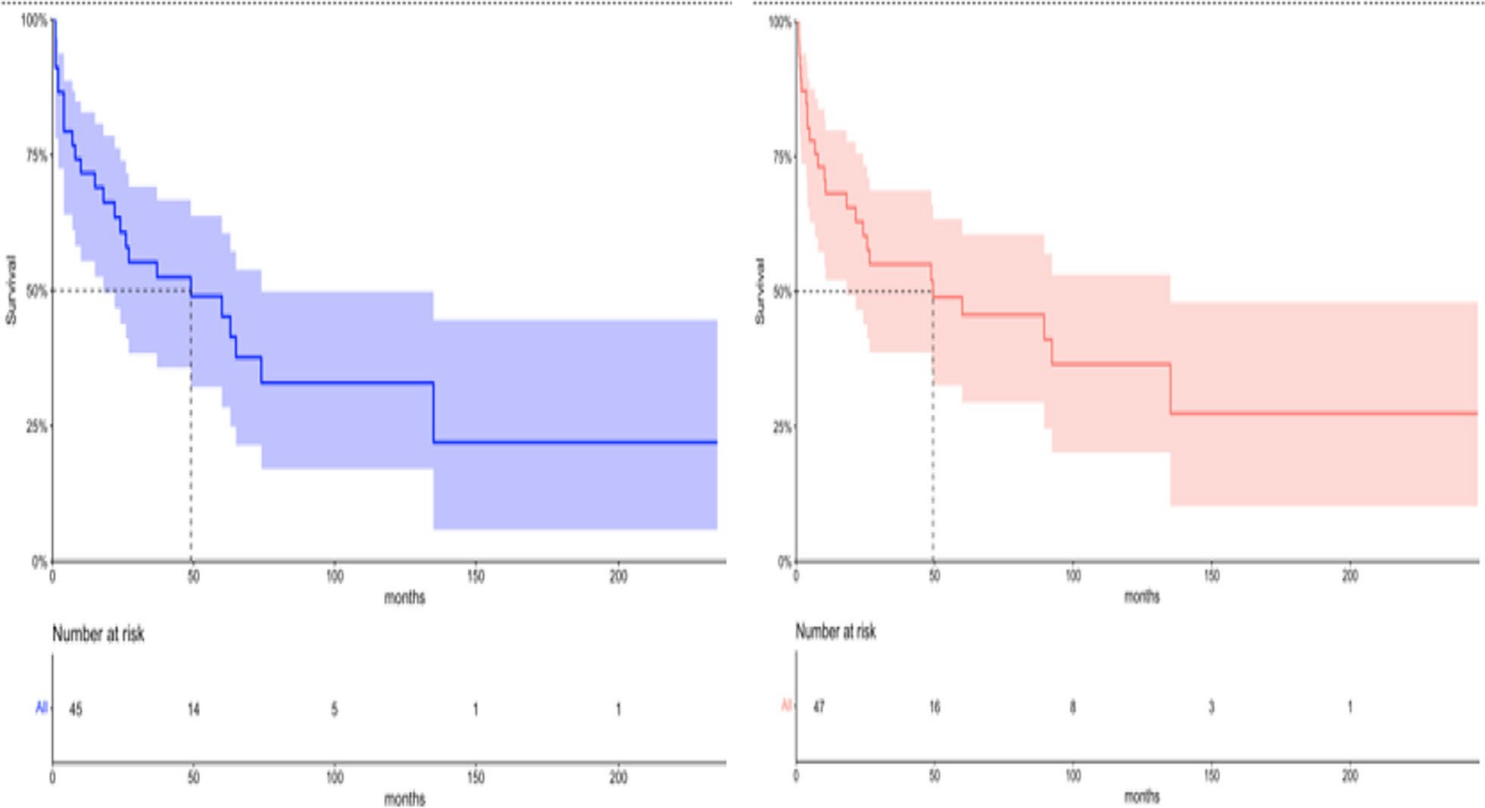
PFS (**A**) and OS (**B**) for 47-Brazilian patients with PCNSL included in the study

The median follow-up was 24.4 months (95% CI: 10.2–41.0 months), with a median time between diagnosis and the beginning of therapy of 34 days (95% CI: 29–46 days). The median overall survival was 49 months (95% CI: 18–74 months), with an estimated 2-year OS of 60.2% (95% CI: 47.0–77.1%) and 45.5% (95% CI: 32.1–64.7%) at 5-year [Fig. 2]. The median progression-free survival was 37 months (95% CI: 15–65 months), with an estimated 2-year PFS of 59.2% (95% CI: 45.8–76.6%) and 36.3% (95% CI: 23.0–57.1%) at 5-year [Fig. 2].

**Fig. 3:**
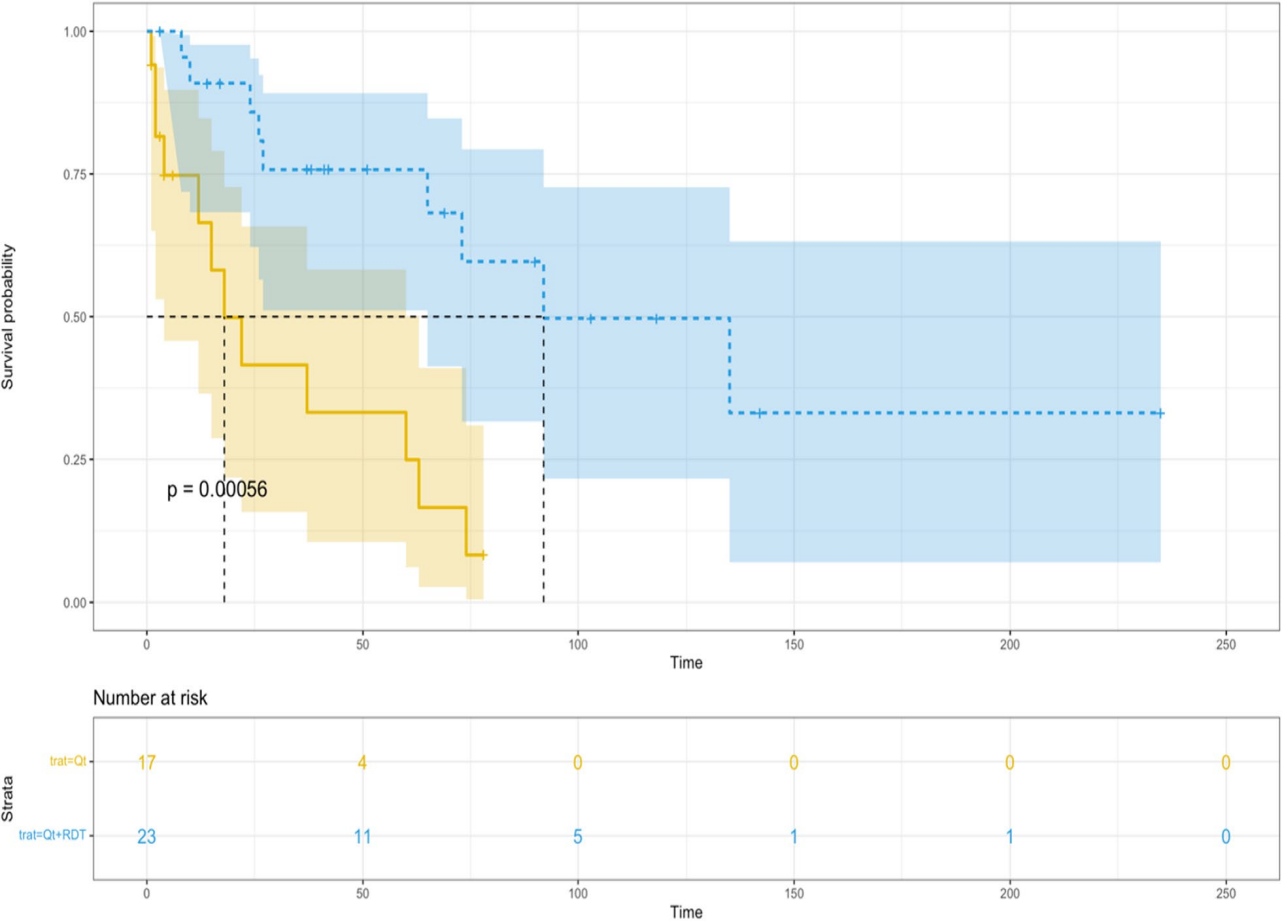
OS curves for PCNSL patients treated with HD-MTX-based therapy followed by WBRT plus HIDAC (blue-line) versus HD-MTX-based induction followed by HIDAC alone (yellow-line)—*p* < 0.001

The median OS was 92 months (95% CI: 65 months—not reached) for patients treated with HD-MTX followed by combined consolidation therapy using WBRT plus HIDAC [N = 23/40] and just 18 months (95% CI: 4–63 months) for those consolidated only with HIDAC [N = 17]. The estimated 2-year OS was 85.8% (95% CI: 72.2–100%) for CCT (WBRT plus HIDAC) and 41.5% (95% CI: 21.7–79.7%) for HIDAC alone (*p* < 0.001) (Fig. 3).

### Prognostic factors

In univariate analysis, the variables associated with increased OS were age < 60 years [HR: 0.46, 95% CI: 0.20–0.97, *p* = 0.05], Hb ≥ 120 g/L [HR: 0.84, 95% CI: 0.71–0.99, *p* = 0.06] and consolidation with WBRT [HR: 0.20, 95% CI: 0.09–0.47, p < 0.001]. LDH ≥ UVN [HR: 2.03, 95% CI: 1.08 -3.79, p = 0.02], hypoalbuminemia (< 3.5 g/dL) [HR: 3.39, 95% CI: 1.37–8.42, *p*  = 0.009], ECOG ≥ 2 [HR: 9.35, 95% CI: 1.10–19.10, *p* = 0.04], KPS < 70 [HR: 2.43, 95% CI: 0.89–6.61, *p* = 0.05] and intermediate-risk or high-risk Nottingham/Barcelona prognostic score [HR: 4.39, 95% CI: 1.33- 14.4, *p* = 0.01] were associated with decreased OS.

Gender (*p* = 0.45), β2-microglobulin ≥ UVN (*p* = 0.41), clinical stage IV/multifocal disease (*p* > 0.99), IELSG score (*p* > 0.99), MSKCC score (*p* = 0.12), CSF protein > 40 mg/dL (*p* = 0.67), deep brain involvement (*p* = 0.22), orbit/eyeball involvement (*p* = 0.11), cranial nerve impairment (*p* = 0.10), neutrophilia (*p* = 0.61), lymphopenia (*p* = 0.50), monocytosis (*p* = 0.88), thrombocytopenia (*p* = 0.34), non-GCB phenotype (*p* = 0.08), CD10 expression (*p* = 0.63) and CD30 expression (*p* = 0.87) did not show statistically significant association with OS in this cohort, at 95% significance.

Age < 60 years [HR: 0.43, 95% CI: 0.19–0.97, *p* = 0.04] was associated with increased PFS, however, ECOG ≥ 2 [HR: 9.88, 95% CI: 1.22–8.00, *p* = 0.03] and intermediate/high-risk IELSG score [HR: 10.6, 95% CI: 1.21–19.80, *p* = 0.03] were associated with decreased PFS. The other variables did not show a statistically significant association with PFS.

## Discussion

In this study, we showed that patients with PCNSL consolidated with WBRT plus HIDAC presented increased OS than patients consolidated with chemotherapy alone (HR: 0.20, 95% CI: 0.09–0.47, *p* < 0.001). The median OS was 92 months for patients consolidated with WBRT plus HIDAC and only 18 months for those receiving only HIDAC (*p* < 0.001). ASCT was not evaluated because no one patient was submitted to this therapy in our cohort.

This study analyzed retrospectively patients with PCNSL diagnosed and treated in a single center from 2000 to 2021. In a period of 21 years, were identified 47 patients and 45% of them were alive at 5-years. This result was similar to previous reports encompassing patients with PCNSL receiving regimens of treatment containing HD-MTX [46]. A Danish retrospective study showed an overall survival of 26% at 5-year for 48 patients with PCNSL treated from 1971 to 1990 [26]. Likewise, an analysis of 3,100 PCNSL cases from the *Surveillance Epidemiology End Results* (SEER) database, treated between 2000 and 2008 demonstrated an estimated 5-year OS of 31.2% [46].

Data from 4158 patients with PCNSL included in the SEER database since 1992 until 2011 showed a 5-year OS of 9% for patients living with HIV and 26.2% for immunocompetent patients [41]. Zeremski et al. [47] also reported 86 patients with PCNSL diagnosed between 2000 and 2015, 20 of them treated in clinical trials and 66 cases in real-life settings. The 5-year OS was 40% for patients enrolled in clinical trials and 23% for those treated in real-life settings [47]. Table 3 summarizes the main cohorts including PCNSL patients and the clinical outcomes reported.

**Table 3:** Main case series and outcomes for PCNSL

Cohort	Period	N	2-year OS (%)	5-year OS (%)
Krogh-Jensen et al. [26] (Denmark)	1971–1990	48	38	26
Villano et al. [46] (SEER, USA)	2000–2008	3100	42.6	31.2
Shiels et al. [41] (SEER, USA)	1992–2011	4158	–	HIV (9.0) Non-HIV (26.2)
HC-FMUSP (2022) (Brazil)	2000–2021	47	60.2	45.5
Zeremski et al. [47] (Germany)	2000–2015	86 CT (20) RL (66)	65 37.9	40 23

*OS* overall survival, *CT* clinical trial, *RL* “real-life” settings

PCNSL is rare and despite the advances obtained in the last decade it prognosis remains dismal. Moreover, its treatment is usually supported by low level of scientific evidence, because the majority of the trials involving this subtype of NHL included small number of patients. Prospective and randomized studies with an appropriate number of cases are scarce. Although the absence of a standard regimen of treatment, its therapy involves a first phase of induction combining HD-MTX as backbone with others antineoplastic agents followed by a second phase of consolidation for patients acquiring complete remission or partial remission after the induction phase. The main anticancer drugs added to HD-MTX are vincristine, procarbazine, temozolomide, HIDAC and lomustine [1, 31, 35]. Most of them are able to cross the blood–brain to penetrate into the CNS tissue [31, 35].

A consolidative strategy has been also added to the PCNSL treatment because it improves disease control, reducing the chance of early-relapses and eventually providing long-term responses [18, 29]. In the same way as for the induction phase, the best strategy for PCNSL consolidation is still undefined. However, the most common alternatives involve high-doses (45 Gy) of whole brain irradiation (WBRT) and autologous hematopoietic stem cell transplantation (ASCT) [14, 18].

In fact, WBRT has been introduced as a consolidative approach for PCNSL since these tumors are highly sensitive to irradiation [18, 29, 47]. Isolated WBRT for PCNSL produces high rates of overall response and a median OS of 10–18 months, but only 5% of patients will be alive in 5 years [27, 40]. Hence, isolated WBRT is currently considered a palliative approach, because it is associated with higher rates of disease recurrence. However, when used for consolidate patients in CR or PR after HD-MTX-based chemotherapy, WBRT improves the chance of overall survival at 5-years in almost 50% [18, 29, 47]. In a prospective study, involving 31 patients with PCNSL, Bessell et al. [6], showed that the omission of WBRT or its dose reduction from 45 to 30 Gy was associated with higher rates of lymphoma recurrence and decreased overall survival [6].

WBRT following HD-MTX-based therapy is still controversial because of the high rates of neurological toxicity and cognitive impairment associated with this strategy. [19, 22]. However, Ferreri et al. [15] demonstrated that consolidation with WBRT in total-dose of 36 Gy did not reduce survival and was well tolerated in patients with PCNSL in CR after HD-MTX-based induction. Higher doses did not change the outcomes and could increase the risk of neurotoxicity [15]. On the other hand, ASCT has been shown to be a better alternative than WBRT for consolidation of PCNSL patients, as it is not highly associated with neurological toxicity [12]. In that regard, Soussain et al. [43], demonstrated a rate of 60% of complete response, and a median OS and PFS of 58 months and 41 months, respectively, in 43 relapsed or refractory PCNSL patients submitted to ASCT. In this study, there was 7% of mortality related to transplant [43].

However, the best results of ASCT as consolidation in PCNSL were obtained with incorporation of the alkylating agent thiotepa in the conditioning regimen [12, 25, 45]. A multicenter study involving 81 PCNSL patients consolidated with ASCT using thiotepa in the conditioning regimen after HD-MTX-based induction, showed 77.2% of complete remission and 5% of transplant-related mortality [24]. However, ASCT is not widely free of side effects and long-term toxicities [42]. Moreover, the incidence of PCNSL is more prevalent in elderlies, where multiples comorbidities may prevent indication of ASCT because of higher risk of complications related to treatment in this context [11].

To solve many issues involving the treatment of PCNSL, the IELSG group designed a randomized phase-2 study (IELSG-32) enrolling 219 PCNSL patients from 53 European centers comparing three therapies of induction and two regimens of consolidation. The authors found that HD-MTX, cytarabine, thiotepa and rituximab (Matrix) was associated with superior PFS and OS in comparison to HD-MTX plus cytarabine and HD-MTX plus cytarabine and rituximab. CR rate was 49% for Matrix regimen, 30% for HD-MTX plus cytarabine and rituximab and only 23% for HD-MTX plus cytarabine. Hematological toxicities grade 3 or 4 were also higher for patients in the Matrix arm, but the rate of infection was similar in the three groups [12].

Patients acquiring CR or PR after induction were later randomized for WBRT or ASCT in IELSG-32. There was no statistical significant difference between them, the overall survival was 85% at 2-years in the WBRT arm and 71% for ASCT, *p* = 0.17. At 2-years, PFS was 80% for WBRT and 69% for ASCT, *p* = 0.12. Cognitive dysfunctions were more common with WBRT. However, the authors suggested that both strategies were feasible and effective to consolidate PCNSL patients in first CR or PR [14].

Unfortunately, as our study was performed retrospectively, we were not able to apply specific scales and instruments to assess mild cognitive abnormalities related to WBRT. It may justify the low frequency of cognitive impairment associated with CNS radiotherapy in our cohort. On the other hand, as described above, ASCT may also provokes acute and chronic damages. As an example, the Nordic group recently reported that among 271 patients with lymphoma submitted to ASCT, 98% of cases had at least one moderate or severe late adverse event and 56% presented at least one long-term life-threatening complication [42]. Moreover, the IELSG-32 study showed that the risk of brain lymphoma progression was 1.5 times higher for ASCT than WBRT. Likewise, the risk of death was 1.67 times superior in the ASCT arm than for WBRT group [14].

To reduce the morbidity associated with brain irradiation some authors proposed dose reduction for patients in CR. The hyper-fractionation of the planned total dose also can reduce the neurotoxicity [15, 24, 39, 40]. Currently, 45 Gy WBRT is not a standard of care for PCNSL treatment. In the IELSG-32 study, the authors employed 36 Gy WBRT and a 9 Gy boost dose was applied in patients with partial response, with satisfactory outcomes [14]. Use of low-dose WBRT (ld-WBRT) with 23.4 Gy (13 fractions of 180 cGy) also appears to be a promising strategy, and it was recently evaluated in a phase II trial that compared the R-MPV-A regimen (rituximab, HD-methotrexate, procarbazine, vincristine and cytarabine) with and without ld-WBRT in 91 newly diagnosed PCNSL patients. Preliminary results showed a benefit of PFS (2-year PFS 78 vs. 54%) without increasing neurotoxicity [34].

In general, the current literature indicates that WBRT and ASCT offer similar PFS for PCNSL patients, although only a few randomized trials have compared both strategies head to head [14, 23]. In 2019, a French multicenter consortium reported the results of 140 PCNSL patients treated in 23 distinct centers. The authors found no statistical significant difference between WBRT and ASCT, and both were effective and well tolerated for patients under 60 years [23].

Although neurotoxicity dos not favor WBRT, recent studies appointed several issues associated with ASCT and many obstacles to its use in PCNSL. Among the later are patient’s advanced age, usual low performance status, socioeconomic issues, and restricted access to thiothepa for conditioning, particularly in low-income countries. Therefore, WBRT is still considered an attractive, effective, and relatively safe option for PCNSL consolidation, especially when approaches to reduce its toxicity are applied, such as load reduction, dose fractionation and frequent monitoring of possible adverse effects.

In agreement with previous reports, in univariate analysis we showed that age above 60 years, high LDH levels, hemoglobin below 120 g/L, albumin inferior to 3.5 g/dL, poor performance status (ECOG ≥ 2) and no use of WBRT as consolidation were predictive of poor outcomes in the Brazilian cohort. Corry et al. [10] also analyzed 62 patients with PCNSL treated between 1982 and 1994 and found that age > 60 years, ECOG > 1 and WBRT inferior to 45 Gy were predictors of decreased overall survival in univariate analysis [10]. Similarly, Ferreri et al. [13] conducted a retrospective and multicenter study enrolling 378 PCNSL patients followed from 1980 to 2000 from IELSG registry. The authors found that age > 60 years, ECOG ≥ 2, high LDH, high concentration of protein in CSF and involvement of deep brain structures were independently associated to poor survival in PCNSL applying multivariate analysis [13]. Likewise, in a study from the *Memorial Sloan Kettering Cancer Center* (MSKCC), where 338 PCNSL patients followed between 1983 and 2003 were analyzed, age > 50 years and KPS < 70 were predictive of decreased overall survival [3]. Our findings confirmed the prognostic value of the previously mentioned variables and identified new potential biomarkers associated with dismal prognosis in PCNSL, such as the occurrence of anemia and hypoalbuminemia.

## Conclusion

In this real-life study of 47 PCNSL patients treated in a Brazilian single center we found similar clinical characteristics and prognostic factors among our cohort and previous reports. Consolidation with WBRT plus HIDAC showed better outcomes than chemotherapy alone following a HD-MTX-based induction phase. For patients with PCNSL living in low-income countries with no universal access to autologous stem cell transplantation and thiotepa, WBRT should not be seen as “enemy” but as “friendly approach”. In summary, WBRT remains an interesting, efficient and relatively safe option to consolidate PCNSL patients in CR or PR after induction with HD-MTX therapy, particularly in resource-constrained settings.

## Data Availability

All data generated and analyzed during this study were included in this published article. The raw data for this study are in possession of the correspondence author and may be fully available in the event of a request to the correspondence author via e-mail.

## References

[1] Abrey LE, Yahalom J, DeAngelis LM (2000). Treatment for primary CNS lymphoma: the next step. J Clin Oncol.

[2] Abrey LE, Batchelor TT, Ferrei AJM, Gospodarowicz M, Pulczyinski EJ, Zucca E (2005). Report of an international workshop to standardize baseline evaluation and response criteria for primary CNS lymphoma. J Clin Oncol.

[3] Abrey LE, Ben-Porat L, Panageas KS, Yahalom J, Brekey B, Curran W (2006). Primary central nervous system lymphoma: the Memorial Sloan-Kettering Cancer center prognostic model. J Clin Oncol.

[4] Akhter A, Masir N, Elyamany G, Phang KC, Mahe E, Al-Zahrani AM (2015). Differential expression of Toll-like receptor (TLR) and B-cell receptor (BCR) in primary diffuse large B-cell lymphoma of the central nervous system. J Neurooncol.

[5] Bessell EM, Graus F, Lopez-Guillermo A, Lewis SA, Villa S (2004). Primary non-Hodgkin’s lymphoma of the CNS treated with CHOD/BVAM or BVAM chemotherapy before radiotherapy: long-term survival and prognostic factors. Int J Radiat Oncol Biol Phys.

[6] Bessell EM, López-Guillermo A, Villá S, Verger E, Nomdedeu B, Petit J (2002). Importance of radiotherapy in the outcome of patients with primary CNS lymphoma: an analysis of the CHOD/BVAM regimen followed by two different radiotherapy treatments. J Clin Oncol.

[7] Braggio E, Van Wier S, Ojha J, McPhail E, Asmann YW, Egan J (2015). Genome-wide analysis uncovers novel recurrent alterations in primary central nervous system lymphomas. Clin Cancer Res.

[8] Brandsma D, Bromberg JEC (2018). Primary CNS lymphoma in HIV infection. Handb Clin Neurol.

[9] Calimeri T, Steffanoni S, Gagliardi F, Chaira A, Ferreri AJM (2021). How we treat primary central nervous system lymphoma. ESMO Open.

[10] Corry J, Smith JG, Wirth A, Quong G, Liew KH (1998). Primary central nervous system lymphoma: age and performance status are more important than treatment modality. Int J Radiat Oncol Biol Phys.

[11] Dahi P, Lee J, Devlin SM, Ruiz J, Maloy M, Rondon-Clavo C (2021). Toxicities of high-dose chemotherapy and autologous hematopoietic cell transplantation in older patients with lymphoma. Blood Adv.

[12] Ferreri AJ, Cwynarski K, Pulczynski E, Pozoni M, Deckert M, Politi LS (2016). Chemoimmunotherapy with methotrexate, cytarabine, thiotepa, and rituximab (MATRix regimen) in patients with primary CNS lymphoma: results of the first randomisation of the International Extranodal Lymphoma Study Group-32 (IELSG-32) phase 2 trial. Lancet Hematol.

[13] Ferreri AJM, Blay JY, Reni M, Pasini F, Spina M, Ambrosetti A (2003). Prognostic scoring system for primary CNS lymphoma: the International Extranodal Lymphoma Study Group experience. J Clin Oncol.

[14] Ferreri AJM, Cwynarski K, Pulczynski E, Fox CP, Schorb E, Rosée PL (2017). Whole-brain radiotherapy or autologous stem cell transplantation as consolidation strategies after high-dose methotrexate-based chemoimmunotherapy in patients with primary CNS lymphoma: results of the second randomization of the International Extranodal Lymphoma Study Group-32 phase-2 trial. Lancet Hematol.

[15] Ferreri AJM, Verona C, Politi LS, Chiara A, Perna L, Villa E (2011). Consolidation radiotherapy in primary central nervous system lymphoma: imapct on outcome of different fields and doses in patients in complete remission after upfront chemotherapy. Int J Radiat Oncol Biol Phys.

[16] Ferreri AJM (2017). Therapy of primary central nervous system lymphoma: role of intensity, radiation, and novel agents. Hematology Am Soc Hematol Educ Program.

[17] Fukumura K, Kawazu M, Kojima S, Ueno T, Sai E, Soda M (2016). Genomic characterization of primary central nervous system lymphoma. Acta Neuropathol.

[18] Grommes C, DeAngelis LM (2017). Primary CNS lymphoma. J Clin Oncol.

[19] Grommes C, Rubenstein JL, DeAngelis LM, Ferreri AJM, Batchelor TT (2019). Comprehensive approach to diagnosis and treatment of newly diagnosed primary CNS lymphoma. Neuro Oncol.

[20] Han CH, Batchelor TT (2017). Diagnosis and management of primary central nervous system lymphoma. Cancer.

[21] Hans CP, Weisenburger DD, Greiner TC, Gascoyne RD, Delabie J, Ott G (2004). Confirmation of the molecular classification of diffuse large B-cell lymphoma by immunohistochemistry using a tissue microarray. Blood.

[22] Hoang-Xuan K, Bessell E, Bromberg J, Hottinger AF, Preusser M, Rudà R (2015). Diagnosis and treatment of primary central nervous system lymphoma in immunocompetent patients: guidelines from the European Association for Neuro-Oncoloy. Lancet Oncol.

[23] Houillier C, Taillandier L, Dureau S, Lamy T, Laadhari M, Chinot O (2019). Radiotherapy or autologous stem-cell transplantation for primary CNS lymphoma in patients 60 years of age and younger: results of the intergroup ANOCEF-GOELAMS randomized phase II PRECIS study. J Clin Oncol.

[24] Illerhaus G, Kasenda B, Ihorst G, Egerer G, Lamprecht M, Keller U (2016). High-dose chemotherapy with autologous haemopietic stem cell transplantation for newly diagnosed primary CNS lymphoma: a prospective, single-arm, phase 2 trial. Lancet Hematol.

[25] Kokolo MB, Fergusson D, O’Neill J, Tay J, Tinmouth AT, Stewart D (2014). Effectiveness and safety of thiotepa conditioning treatment prior to stem cell transplant in patients with central nervous system lymphoma. Leuk Lymphoma.

[26] Krogh-Jensen M, D’Amore F, Jensen MK, Christensen BE, Thorling K, Pedersen M (1995). Clinicopathological features, survival and prognostic factors of primary central nervous system lymphoma: trends in incidence of primary central nervous system lymphoma and primary malignant brain tumors in a well-defined geographical area. Population-based data from the Danish Lymphoma Registry, LYFO, and Danish Cancer Registry. Leuk Lymphoma..

[27] Littman P, Wang CC (1975). Reticulum cell sarcoma of the brain. A review of the literature and a study of 19 cases. Cancer.

[28] MacMahon EM, Glass JD, Hayward SD, Mann RB, Becker PS, Charache P (1991). Epstein-Barr virus in AIDS-related primary central nervous system lymphoma. Lancet.

[29] Mendez JS, Grommes C (2018). Treatment of primary central nervous system lymphoma: from chemotherapy to small molecules. Am Soc Clin Oncol Educ Book.

[30] Montesinos-Rongen M, Terrao M, May C, Marcus K, Blümcke I, Hellmich M (2021). The process of somatic hypermutation increases polyreactivity for central nervous system antigens in primary central nervous system lymphoma. Haematologica.

[31] Morris PG, Correa DD, Yahalom J, Raizer JJ, Schiff D, Grant B (2013). Rituximab, methotrexate, procarbazine, and vincristine followed by consolidation reduced-dose whole-brain radiotherapy and cytarabine in newly diagnosed primary CNS lymphoma: final results and long-term outcomes. J Clin Oncol.

[32] Nakamura T, Tateishi K, Niwa T, Matshushita Y, Tamura K, Kinoshita M (2016). Recurrent mutations of CD79b and MYD88 are the hallmark of primary central nervous system lymphomas. Neuropathol Appl Neurobiol.

[33] Omuro A, Correa DD, DeAngelis LM, Moskowitz CH, Matasar MJ, Kaley TJ (2015). R-MPV followed by high-dose chemotherapy with TBC and autologous stem-cell transplant for newly diagnosed primary CNS lymphoma. Blood.

[34] Omuro A, DeAngelis LM, Karrison T, Bovi JA, Rosenblum M (2020). Randomized phase II study of rituximab, methotrexate (MTX), procarbazine, vincristine, and cytarabine (R-MPV-A) with and without low-dose whole-brain radiotherapy (LD-WBRT) for newly diagnosed primary central nervous system lymphoma (PCNSL). J Clin Oncol.

[35] Morris PG, Correa DD, Yahalom J, Raizer JJ, Schiff D, Grant B (2013). Rituximab, Methotrexate, Procarbazine, and Vincristine followed by consolidation reduced-dose whole-brain radiotherapy and cytarabine in newly diagnosed primary CNS lymphoma: final results and long-term Outcome. J Clin Oncol.

[36] Portegies P, Corssmit N (2000). Epstein-Barr virus and the nervous system. Curr Opin Neurol.

[37] Reis DGC, Levy D, Lage LAPC, Culler HF, Rocha V, Bydlowski SP (2021). New genetic prognostic biomarkers in primary central nervous system lymphoma (PCNSL). Brain Behav.

[38] Sethi TK, Reddy NM (2019). Treatment of newly diagnosed primary central nervous system lymphoma: current and emerging therapies. Leuk Lymphoma.

[39] Shah GD, Yahalom J, Correa DD, Lai RK, Raizer JJ, Schiff D, LaRocca R (2007). Combined immunochemotherapy with reduced whole-brain radiotherapy for newly diagnosed primary CNS lymphoma. J Clin Oncol.

[40] Shibamoto Y (2013). Radiation therapy for primary central nervous system lymphoma. Oncol Rev.

[41] Shiels MS, Pfeiffer RM, Besson C, Clarke CA, Morton LM, Nogueira L (2016). Trends in primary central nervous system lymphoma incidence and survival in US. Br J Haematol.

[42] Smeland K, Holte H, Fagerli UM, Bersvendsen H, Hjermstad MJ, Loge JH (2022). Total late effect burden in long-term lymphoma survivors after high dose therapy with autologous stem-cell transplant and its effect on health-related quality of life. Haematologica.

[43] Soussain C, Hoang-Xuan K, Taillandier L, Fourme E, Choquet S, Witz F (2008). Intensive chemotherapy followed by hematopoietic stem-cell rescue for refractory and recurrent primary CNS and intraocular lymphoma: Société Française de Greffe de Moëlle Osseuse-Thérapie Cellulaire. J Clin Oncol.

[44] Swerdlow SH, Campo E, Pileri SA, Harris NL, Stein H, Siebert R (2016). The 2016 revision of the World Health Organization classification of lymphoid neoplasms. Blood.

[45] Van der Weyden C, Prince HM (2016). High-dose thiotepa-based conditioning regimens for relapsed lymphoma involving the central nervous system: from “orpham drug” to a standard-of-care?. Leuk Lymphoma.

[46] Villano JL, Koshy M, Shaikh H, Dolecek TA, McCarthy BJ (2011). Age, gender, and racial differences in incidence and survival in primary CNS lymphoma. Br J Cancer.

[47] Zeremski V, Khoeler M, Fisher T, Schalk E (2016). Characteristics and outcome of patients with primary CNS lymphoma in a “real-life”setting compared to a clinical trial. Ann Hematol.

